# Developing expertise, customising sleep, enhancing study practices: exploring the legitimisation of modafinil use within the accounts of UK undergraduate students

**DOI:** 10.1080/09687637.2018.1555231

**Published:** 2019-01-16

**Authors:** Alice Steward, Martyn Pickersgill

**Affiliations:** a Independent, Edinburgh, United Kingdom of Great Britain and Northern Ireland;; b Centre for Biomedicine, Self and Society, Usher Institute, Old Medical School, Teviot Place, University of Edinburgh, Edinburgh, United Kingdom of Great Britain and Northern Ireland

**Keywords:** Modafinil, enhancement, students, UK, cognitive enhancers, pharmaceuticalisation

## Abstract

**Introduction and aim:** Increasing numbers of students are reportedly using prescription medications to enhance cognition. This study aimed to generate qualitative data on UK students’ understandings and perspectives of the risks and benefits surrounding so-called ‘study drugs’ (particularly, modafinil).

**Design and methods:** Fifteen undergraduate students studying biomedical science subjects were interviewed about their perspectives on study drugs. Interviews were recorded and transcribed for thematic analysis. Users and non-users were included in the sample.

**Results:** The prescription status and comparisons to other legal and illicit stimulants informed accounts of the (lack of) risks associated with study drugs, legitimising use. The customisation of sleep(iness) and wakefulness was described as a key benefit of study drug use. Drivers of use related to university pressures and desires to increase productivity. In periods of heightened stress, such as examinations, students reported altered practices and perspectives on risk.

**Discussion and conclusions:** We noted the contextual nature of students’ use and risk appraisals, with fluctuating social contexts and pressures over time being capable of altering prior assessments and current practices (including the legitimisation of study drug consumption). Further, we highlighted the degree to which students leveraged their biomedical and experiential expertise to account for drug consumption.

## Introduction

Modafinil and Adderall are psychostimulants used to treat narcolepsy and attention deficit hyperactivity disorder (ADHD) respectively. Yet, as we know, drugs developed to treat medical categories can be used to enhance aspects of social life (Elliott, 2011). While there is evidence that these drugs elicit cognitive effects in their target populations, far more doubt exists regarding whether they advance cognition in ‘healthy’ populations (Ilieva, Hook, & Farah, 2015). However, the notion that these drugs improve cognition in unaffected individuals is prevalent in the media (Coveney, Nerlich, & Martin, 2009; Forlini & Racine, 2009a), and potentially informs the perceptions of university students. Stimulants have been described as ‘study drugs’, and used during, for instance, periods of high stress (e.g. exams and deadlines) (Hildt, Lieb, & Franke, 2014). In such situations, pharmaceuticals have been employed to improve academic performance through increased concentration, alertness, and levels of comprehension (DeSantis, Noar, & Webb, 2010; Vrecko, 2013).

While the medical use of modafinil has been restricted even further following a revised risk-benefit profile, suggesting deleterious side-effects, students’ use is allegedly increasing (DeSantis, Webb, & Noar, 2008; European Medicines Agency, 2010). Some commentators have regarded non-prescription uses of pharmaceuticals as study drugs as dangerous and necessitating action (McCabe, Knight, Teter, & Wechsler, 2005; White, Becker-Blease, & Grace-Bishop, 2006). Usage has thus become highly debated, though - as Racine, Rubio, Chandler, Forlini, and Lucke (2014) point out - analyses of this can be rather speculative. Analysis without interrogation of students’ own accounts of study drug (non-)use necessarily comes with assumptions about perceptions and practices that could be distant from lived experience (Ketchum, 2013; Morrison, 2015; Pickersgill & Hogle, 2015). Consequently, it is imperative to localise evaluations of student practices within the specific contexts in which they occur and to consider wider sociocultural environments (Shook, Galvagni, & Giordano, 2014). Through considering the specific contexts, values, and understandings that underlie students’ consumption practices (including a lack of consumption) and discourses of legitimation, qualitative studies can contribute actual and potential user perspectives and experiences to policy and ethical debates.

This paper draws on data obtained from qualitative interviews with UK undergraduates, and seeks to expand current understanding of how university students perceive and negotiate the risks and benefits of study drugs, particularly modafinil. It seeks to contribute to debates regarding how students legitimise and account for their use (or not) of study drugs, and how patterns of (contingent) consumption are presented as relating to their shifting educational contexts.

## Background

Our analysis contributes to empirical studies of study drug use. It also relates to sociological and other social scientific writings on lay knowledge of biomedical substances and practices, and recent debates about pharmaceuticalisation; i.e. ‘the translation or transformation of human conditions, capabilities, and capacities into opportunities for pharmaceutical intervention’ (Williams, Martin, & Gabe, 2011: 711). Below, we introduce each of these literatures, in turn, dwelling in particular on prior work on study drug use.

In the US, Dupont, Coleman, Bucher, and Wilford (2008) found that 5.3% of US students they surveyed had made use of the stimulant methylphenidate (e.g. Ritalin) for nonmedical reasons at least once. This included for recreational reasons. Further US research from DeSantis et al. (2008) found that of 1811 undergraduates surveyed, 34% reported illicitly consuming prescription stimulants. Different US studies have also highlighted similar means of accounting for the use of prescription stimulants, e.g. via legitimating discourses that minimise risk including through comparison with legally-obtainable substances (Cutler, 2014; DeSantis & Hane, 2010). National differences in actual practices of ‘study drug’ use, however, imply the need for case studies across nations. For instance, Forlini, Schildmann, Roser, Beranek, and Vollmann (2015) noted that despite widespread knowledge of prescription stimulants for the purposes of cognitive enhancement, only 2.2% of their sample of German university students consumed these. In a survey reported by Singh, Bard, and Jackson (2014: 1), a ‘substantial majority of students in the UK and Ireland were unaware of and/or uninterested’ in consuming modafinil, methylphenidate or Adderall for purposes of augmenting cognition.

Though emerging evidence suggests differences in the incidence of consumption between different cultural contexts, there do seem to be some key similarities in terms of the nature of drug use. Specifically, Forlini and colleagues found that exams ‘and competitive situations were predominant motivators of use’ (Forlini et al., 2015: 83; Forlini & Racine, 2009b). Eickenhorst, Vitzthum, Klapp, Groneberg, and Mache (2012) and Hildt et al. (2014) obtained similar results in Germany. In the US, DeSantis et al. (2010) found too that stimulants were leveraged to manage ‘periods of high academic stress’, for purposes of both reducing fatigue and enhancing cognition (see also Kolar, 2015). Qualitative and survey data obtained by Hupli, Didžiokaitė, and Ydema (2016) indicate similar patterns for university students in Lithuania and the Netherlands, and likewise Partridge, Bell, Lucke, and Hall (2013) for Australian students.

Gabe and Bury (1996: 74) have noted that ‘everyday life comprises knowledgeable people assimilating large amounts of technical information about risk’. This includes information about the illicit or off-label use of pharmaceuticals and other chemical entities for the purposes of pleasure or performance enhancement. In the case of study drugs, prior research proposes students view prescription stimulants as less harmful than illicit ‘hard’ drugs (Judson & Langdon, 2009). This perception rests on the prescription status of drugs, symbolising medicine’s endorsement of their safety (Looby, Kassman, & Earleywine, 2014). Further, concerns have arisen over the extent to which users are informed of, and appreciate, the risks (DeSouza, 2015; Ragan, Bard, Singh, & Independent Scientific Committee on Drugs, 2013). In sum, there could be reasons to think that the ‘lay pharmacology’ (Webster, Douglas, & Lewis, 2009) of study-drug users is significantly different to the credentialled pharmacological knowledge of biomedical experts.

Despite these worries, evidence from case studies beyond the realms of study drugs suggest that different kinds of expertise about study drugs could be produced and expressed by users, and put to various ends (e.g. justifying, moderating, and curtailing use). As Hall, Grogan, and Gough (2016) have shown for the case of bodybuilders’ use of synthol, pharmaceutical discourses can be engaged with and leveraged to develop and perform credibility and legitimacy. Bancroft and Reid (2016) have also illustrated how scientific knowledge about illegal drugs is developed and deployed on internet user forums to discuss product quality. Such expertise is not only displayed to others, but can also form part of how the use actually occurs. In research by Rönkä and Katainen (2017), the discourse of internet forum users indicates how scientific and experiential expertise contributes to developing ‘pharmaceutical competences’ (Rönkä & Katainen, 2017: 62) and is brought to bear on consumption practices. Penn (2014) has illuminated how experiential knowledge of the effects of illegal drugs on bodies can also be leveraged to intervene in political campaigns that in Canada have reshaped legal access to cannabis. It is thus important to attend to if, and how, university students assemble and/or deploy different kinds of knowledge to account for their consumption of study drugs, not least given the existence of research which indicates a lack of risk-awareness and formal pharmacological understanding on the part of users (DeSouza, 2015; DeSantis et al., 2010).

Finally, Robitaille and Collin (2016) have noted the utility of pharmaceuticalisation theory for considerations of study drug use. As indicated above, consumption practices have been viewed as adaptive responses to the intensified competitiveness of educational environments and wider sociocultural demands (Hogle, 2005; Vargo & Petroczi, 2016). Coveney (2014) has also shown that drugs are in fact sometimes used to promote sleep, for instance when work demands make ‘natural’ sleep challenging. Accordingly, both wakefulness and sleep can be subject to pharmaceutical intervention, pointing to the complex and dynamic nature of pharmaceuticalisation in contemporary societies (Gabe, Coveney, & Williams, 2016; see also Cloatre & Pickersgill, 2014). This dual role for pharmaceuticals indicates too how, today, sleep is for many people highly ‘customisable’ (Williams, Coveney, & Gabe, 2013). Our data represent a timely means of appraising how modafinil is being assimilated into the array of techniques used to customise sleep as part of a potential pharmaceuticalisation of higher education.

## Methodology

This study employed qualitative, semi-structured interviews to examine students’ understandings and practices surrounding the use of non-prescription drugs for study-related purposes. Following ethics approval, audio-recorded interviews were conducted with 15 students at a UK Russell Group university. Undergraduates, rather than postgraduates, were sampled since they are most frequently depicted as the users of study drugs (Coveney, Gabe, & Williams, 2011). Students in their later years of study were purposively recruited, since they are able to reflect on shifting experiences and workloads over their time at university. Recruitment was achieved through an email invitation to all students taking selected biomedical sciences-related degrees, and only such students were included in the eventual (convenience) sample. Interviews were conducted by AS, who was an undergraduate at the time of the study; this enhanced rapport and generated rich dialogue. The median interview duration was 40 minutes (one short interview lasting only 30 minutes nevertheless provided useful data).

Modafinil was selected as the focal pharmaceutical for the interviews, since unlike Ritalin and Adderall (class B drugs and illegal to possess) it is not illegal to buy in the UK (HM Government, 2017). Given this legal position, we assumed students would be more willing to agree to discuss it than prohibited drugs. Interviews covered a variety of matters, and particularly student experiences of their degree to-date (especially, workload), their familiarity with peers who took study drugs, their (assumed) motivations for use, the circumstances in which use occurred, and issues around side-effects and safety. Patterns of study drug use emerged during the interviews; the sample comprised both users (*n* = 10 [3 male, 5 female]) and non-users (*n* = 5 [all female]). All users took modafinil, with some also having tried Adderall and Ritalin. By recruiting both users and non-users, we aimed to cast greater light on what Coveney in her study of non-users’ expectations about modafinil referred to as ‘the cultural resources, norms, and values that are drawn on to evaluate the acceptability of new and emerging technologies [of enhancement]’ (Coveney, 2011). The same topic guide formed the basis of interviews with both users and non-users.

Thematic analysis was used to inductively interrogate transcribed data, memos, and a research diary kept by Bryman (2008). Transcripts and research notes were thoroughly read numerous times to identify emerging concepts, with analytic memos made alongside this coding process. Data were organised into themes, and then arranged under six meso-level themes: students’ information-seeking practices; benefits of use; associated risks; study drug use in relation to illegal drug use; pressures of university life; and, changing risk assessments in response to contextual shifts. These six themes were amalgamated into two overarching themes that encapsulated the data relevant to the research questions: students’ understandings of study drugs, and decision-making around consumption.

## Results

### Substantiating safety

Overall, students’ understandings of prescription stimulants accorded with the popular perception that these are a relatively safe means of improving academic performance. In this section, we analyse how modafinil especially was legitimised as a ‘safe’ drug with limited side-effects. We consider in particular the role of contrasts and comparisons in constructing this pharmaceutical as a (generally) safe substance.

While previous studies have focussed on the widespread use of pharmaceuticals like Adderall and Ritalin on US campuses (e.g. DeSantis et al., 2010; McCabe et al., 2005), our UK users all declared modafinil as preferable to these stimulants (as well as to recreational drugs). Concerns over addiction and dependency were key components in accounts of these evaluations and appeared to significantly influence perceived risk. Students described how the use of Adderall and Ritalin could result in dependence, and hence these were approached more cautiously. Modafinil was presented as non-addictive, and as producing a ‘less systemic effect’ on the body, with ‘no buzz’. As R1 (user) described: ‘Modafinil is not addictive, that’s why it’s a lot more preferable to other study drugs’.

The side-effects that students did discuss (which were often second-hand accounts) included flushes, headaches, increased thirst, reduced appetite, and interrupted sleep patterns. Overall, these were not considered significant enough to be major deterrents. Other students also presented modafinil as having limited or no side-effects (and both user and non-user perceptions were similar in this regard). R2 (user) reflected: ‘I would say the benefits outweigh the risks; I’ve not had any negative side effects’. R11 (user) described the phenomenology of use as follows: ‘you don’t feel high, you don’t feel like speedy […] that’s why I took it, because it was quite nice and I didn’t feel like I was on drugs’. For this student, modafinil ‘just feels like you took a cup of coffee that lasted the whole day’. This made it more effective for studying, safer, and more pleasant to use. Several students highlighted that side-effects exist for every substance, and those of modafinil may be no worse than commonly used and accepted medications. As one respondent put it, ‘there are side-effects to everything’ (R13, non-user).

Responding to the question ‘how does modafinil compare to other stimulants?’, the risk was often articulated in relation to other study drugs, illicit substances, coffee, and caffeine pills. Coffee was commonly presented as the exemplar of a safe and acceptable means of staying awake. Some students subsequently compared modafinil use to coffee consumption, minimising its significance. Several users, in fact, asserted that the side effects of caffeine were considerably less desirable and less conducive to their goal of maintaining an efficient and lengthy period of study. R1 (user), for instance, noted that modafinil ‘doesn’t make you jitter and shake, not like if you drink too much coffee’. R2 (user) made a more forceful distinction between these substances, arguing that caffeine ‘is definitely more dangerous than modafinil, 100%; you’ve got billions of people addicted to caffeine. It’s so bad for you’. However, some non-users diverged from this comparatively positive and legitimising account of modafinil vs. caffeine. R7 (non-user), for instance, rationalised that the heightened social acceptability and daily use of caffeine were indicators of safety: ‘I drink coffee, and caffeine’s a drug […] but it’s more socially acceptable; it’s legal and it’s everywhere. And it’s not like taking pills; when I’m drinking coffee, it’s like a food – a natural thing’.

The fact that modafinil was taken as a pill was described by one student (R2, user) as implying it should or would be more potent than, for instance, coffee: ‘the whole process of swallowing a pill has more significance, you’re like, okay I’ve taken a pill, therefore, I should be feeling something as it exerts some sort of effect’. The materiality of modafinil thus appeared to hold consequences for perceptions of efficacy, as well as risk: ‘generally, because it’s in pill form, I feel like you might be more careful with it’ (R8, user). While illicit drugs can also come in the form of a pill, for some students this reinforced the status of modafinil as a safe substance. R8 (user), for instance, reflected that ‘I feel like because it comes packaged properly, it should do less damage, versus recreational drugs where you just get who-knows-what from who-knows-who’.

Students largely implied that the prescription status of modafinil increased its safety; after all, it was ‘a medical drug, not a street drug’ (R2, user). Prescription status could also provide a reliable and safe means of determining dosage (for non-medical purpose) and seemed to reinforce perceptions of safety. R1 (user) described this as follows: ‘if you take less than what they recommend on the guidelines for the drug, you assume that you’re not going to experience anything that’s really bad’. These findings underscore the arguments found in Coveney et al. (2011) and Cutler (2014), where trust and authority are invested in medicine and medical institutions to demarcate safe from unsafe pharmaceuticals and drug practices.

In sum, modafinil use was legitimised through constructions of its side-effects as minor, which related to a general presentation of the drug as (comparatively) safe. Similar discourses of minimisation and comparison have been found in some US research (e.g. Cutler, 2014; DeSantis & Hane, 2010). Constructions of safety were substantiated through respondents’ personal experiences (what Webster et al. (2009) might refer to as ‘lay pharmacology’), through comparisons and contrasts (e.g. with other study drugs and caffeine), and through the status of modafinil as a prescription pill. In the latter case, the fact that it came as a tablet also underscored its potential potency. Consequently, the particular materiality of modafinil connects it with the multiple cultural meanings to which pharmaceutical objects can be attached (Martin, 2006).

### Leveraging knowledge

In this section, we want to foreground the different kinds of knowledge that our participants engaged with and leveraged when substantiating their perspective of modafinil and legitimising practices of consumption. To begin with, we continue our discussion of how our participants situated this drug within UK biomedicine, in order to elucidate how understandings of pharmaceutical innovation and regulation played key roles in constructing the ontology of modafinil.

Modafinil’s prescription status denoted a substantial reduction in risk and more favourable attitudes in comparison to illicit drugs (see also Judson & Langdon, 2009; Looby et al., 2014). Students defended their stance through detailing the stringent testing procedures of clinical phase trials that must be met in order for drugs to be approved. Thus, they brought to bear their knowledge about the rigour of pharmaceutical testing and clinical trials in their adjudications of the safety and efficacy of study drugs. In doing this, though, some respondents also destabilised a more generally expressed perception of modafinil as safe for all users. Several students distinguished that testing and approval occurred for use within a specific population and consequently reflected that drugs might elicit different effects in healthy populations. As R3 (user) put it: ‘studies say that they don’t do to your brain what they do to the person they’re designed for, because […] they’re designed to fix something you’re not deficient in’. Relatedly, some students who initially described perceiving modafinil as safe shifted their perception of the drug when discussing its prescription status. Specifically, they described how untested/unapproved applications of drugs could confer additional risks, and that the consequences of these might outweigh any benefits. For R13 (non-user), ‘if you need a prescription for something, the ‘powers that be’ have deemed it more dangerous than just over the counter’.

Contrary to the US students of DeSouza’s (2015) and DeSantis et al. (2010) research, our UK undergraduate respondents articulated various degrees of understanding of the biochemical composition and effects of study drugs. These could be leveraged to legitimise consumption practices. Users, in particular, appeared confident in their research and overall knowledge of the pharmaceuticals they used. Hupli et al. (2016) have proposed that study drug use is not the reckless practice some critics condemn it as; similarly, the students we interviewed asserted that they would not consume drugs without what they considered adequate research. R9 (user) described this as follows:

building up to taking them […] I did quite a lot of research, weighing up the pros and cons and the differences between all types of study drugs that were available to me, to decide which one would suit me best for my personal needs and wants. It wasn’t a spontaneous decision at all.

Students reported different depths of and sources for research, but all concurred that information was abundant and easily accessible. While some turned to Wikipedia, others examined primary peer-reviewed papers to conduct their own analyses of risk-benefit ratios in order to develop what Rönkä and Katainen (2017) call ‘pharmaceutical competences’ that were different in kind to ‘lay pharmacology’ (Webster et al., 2009) constituted through bodily experiences. Several respondents described the scientific nature of their degree as having a role in shaping their research into study drugs: ‘coming from a medical sciences background […] it would be warped of me to not know what I was taking before I started taking it’ (R9, user).

Students that had researched and/or experienced modafinil emphasised how few, if any, side-effects exist, and presented them as minimal. Both this research and wider knowledge of pharmacology were deployed as part of the legitimating repertoires of safety discussed in the previous section, such as by R9 (user): ‘when I was doing research […] I couldn’t see any immediate negative effects from modafinil. The only real concerns were potential sleep deprivation if it’s taken for a long period of time’. Another user, R5, drew on a biomedical idiom (i.e. talk of ‘mechanisms’) to authenticate the constructions of safety via an analogy with coffee (thus employing a similar comparative trope to the respondents discussed above). As they put it: ‘people taking [study drugs] and people chugging coffee, it’s basically the same mechanism […] biologically I’d put them in the same category’.

We can see, then, that several students leveraged and/or claimed different kinds of knowledge about biomedical innovation, pharmaceutical regulation, and neurochemical processes to account for their different perspectives on study drugs. This knowledge did not necessarily pre-exist consideration of drug consumption; rather, dedicated and specific research into the psychoactive properties of study drugs could also shape decision-making about use. In the final section, we turn to a closer examination of how and why modafinil might be consumed by UK undergraduates, paying particular attention to the circumstances under which initial appraisals of the risks and benefits of drug-taking could shift and change in order to (de)legitimise study drug use.

### Enhancing productivity

During the interviews, university life was framed as an environment of heightened competitiveness, resulting in the feelings of stress and anxiety that were described as rife among the sample population (particularly regarding future prospects). Students articulated a range of sources of stress, including self-expectations, familial expectations, time constraints, examination timetables, and financial and/or career considerations. In the words of one respondent: ‘you are up against all these people […] the competition never stops between who gets the best grade, because who gets the best grade gets the better job’ (R13, non-user).

Such pressures were accounted for as legitimising and shaping (potential) study drug use: ‘I don’t think [modafinil] was about in our parents’ generations, but these days everyone is wanting an extra level. I think that’s just our era; the expectations of people have gone up’ (R6, user). Students explained that the convergence of different pressures was significant enough to warrant, and maybe event necessitate study drug use. Clashing examinations was one example given:

some people have really shitty exam schedules […] and if you don’t have study drugs for that, you will not pass your exams, it’s very, very simple […] the university puts you in a position where you feel like you’ve got to do it […] when exam days are like three days apart from each other […] you can’t afford to lose a day because of tiredness […] [study drugs] make sure you don’t crash (R11, user)

Many students linked academic achievement to their self-worth. However, one non-user explained that they did not define their value as a student based on their grades, and consequently felt no compulsion to take study drugs:

if people are placing more emphasis on their grades, then they’re going to want to do it more […] if I don’t do well, it doesn’t define who I am as a person […] but, some people are more willing to do everything like [take study drugs] […] it’s like how far will people go for grades, I feel it’s a bit of a slippery slope (R7, non-user)

All interviewees reported improving academic study as the primary purposes of drug use, particularly valuing improved focus, increased efficiency, and reduced procrastination. These effects were judged highly desirable in the context of time constraints and fatigue approaching exams and deadlines. As one student reflected, a need exists to be ‘more efficient with your time if you are under a lot of pressure, to stay awake for a longer period of time’ (R10, user). This student took modafinil to revise for exams, describing its benefits as follows: ‘I’d have to do ten hours a day and I just can’t sit for that long, and then I took [modafinil] and I did […] it was amazing’.

A demand for efficiency and increased productivity was repeatedly presented as an impetus for and means of legitimising study drug use. Users judged their levels of productivity insufficient and recounted the ability to study for significantly longer periods with the use of drugs. Additionally, modafinil use was described by one student as forming part of a ‘prevention strategy’ to avoid undesirable states of burn-out or exhaustion (which impair students’ ability to work). Specifically, modafinil ‘makes you concentrate more […] you just keep on going because you’re in the zone; it just makes you, the best you on that given day […] giving you more hours in the day to do the stuff you need to do’ (R9, user).

The ability of modafinil to affect wakefulness was well-known, and carefully used to align with students’ desires to customise sleep and sleepiness (Williams et al., 2013) in particular ways. While non-users generally interpreted reduced appetites and inability to sleep as (albeit minimal) negative effects, users often accounted for these as benefits: ‘taking [modafinil] after a certain period of time, you won’t be able to sleep […] it’s one of your uses; you want to increase focus and […] it’s something to stop you falling asleep’ (R1, user). As they further described:

It’s like downing a Redbull [an energy drink] at 3 am, you know you’re not going to sleep […] you feel like crap the next day […] but your assignment will be done. That’s a student’s life; the student makes that cost-benefit decision and if the benefits outweigh the costs, then they’re going to do it (R1, user)

The prudent planning of consumption was presented as a means of avoiding unwanted consequences; for instance, the drug might be consumed later in the day when users strove for an ‘all-nighter’ (i.e. a period of study lasting throughout the night). After all, ‘to do an all-nighter, you’re not going to want to sleep and you also won’t need to eat as well, so that’s good’ (R9, user).

Importantly, these assessments were flexible: they changed with shifting circumstances. Students detailed a kind of threshold of pressure that they felt capable of dealing with; however, exceeding this limit instigated the search for an aid to meet the challenges they faced. An initial risk perception regarding study drugs could thus be overridden by a more pressing need, as illustrated in the extracts below:

I was super against taking them at first…by 2^nd^ year, I was like, that’s fucking stupid, I need to pass my classes (R1, user)

if you’re in that desperate situation where you just need to get it done, then of course you’re going to take something that means you’ll be able to get it done in that time. Because nobody wants to submit something late […] a 5% [reduction in numeric grade] penalty will affect your overall university grade (R9, user)

Students also underscored the influence of drug availability in decision-making. If students found themselves in a situation where drugs were readily obtainable, the potential immediacy of stress resolution could lead students to re-assess their initial stance:

If my friend was sitting in the library opposite me and I had a deadline in two days and was freaking out […] and they said, look, I've got some modafinil, it'll help you concentrate […] I’d still have that reservation, like, that's bad, I shouldn't do that, but also I'd, be, like, that would really help […] it's never been accessible to the point where it's like, okay, I'll just take it, but if I was in that situation, I would definitely be tempted (R4, non-user)

To summarise, study drug users primarily accounted for consumption in terms of work-related goals. Students described study drugs as an effective means of controlling and managing pressures and external stressors encountered in university education (Forlini et al., 2015; Robitaille & Collin, 2016), through customising sleep and sleepiness (Williams et al., 2013). Specifically, users strongly endorsed modafinil’s ability to increase focus, thereby extending the feasible duration of study. The use of study drugs was legitimated as a means of coping with the demands and rigours of university life. Such pressures have also been highlighted in other Australian, European, and US studies (e.g. Eickenhorst et al., 2012; Hildt et al., 2014; Hupli et al., 2016; Jensen, Forlini, Partridge, & Hall, 2016; Kolar, 2015; Partridge et al., 2013). Students’ risk-benefit assessments about study drugs were accounted for as contingent on the magnitude of the aforementioned pressures, and consequently were not fixed. This was apparent in users’ accounts, and in some non-users’ contemplations of scenarios where they would feel impelled to pursue pharmacological coping strategies.

## Conclusion

This article has explored university students’ perspectives around the risks and benefits that accompany ‘study drug’ use, and the knowledge and experiences bought to bear on developing and accounting for these. An important aspect of our research has been the inclusion of both users and non-users: we have demonstrated a resonance between their accounts of why students might consume study drugs, and what the potential harms and advantages are conceived as being.

Users tended to develop pharmaceutical competences (Rönkä & Katainen, 2017), and leveraged pharmacological knowledge to explain that Ritalin and Adderall demonstrate varying levels of addictiveness, but that there is no reported dependence for modafinil. As a consequence of this and the limited side-effects of modafinil (some of which were presented as positives), student users stated that they tended to imbibe that rather than other study drugs. DeSouza (2015) has suggested that undergraduates do not show particular concern for potential risks to study drugs; however, one interpretation of our data is that users negotiate the harms of different study drugs and choose the one that poses the least risk. Another interpretation is that accounts of the risks of other drugs are used as a post hoc justification for the consumption of modafinil, which is a more accessible pharmaceutical in a UK context, as part of a broader strategy of legitimation for study drug use *per se*. Future research should aim to cast greater light on these divergent, but not necessarily mutually exclusive, interpretations.

Underscoring how different meanings can adhere to the same pharmaceutical object (Martin, 2006), the fact that study drugs were present in the form of a pill was received in various ways by different participants in our study, becoming imbricated within different formulations of, and for assessing risk. For example, the fact modafinil came in a pill form could be read as signalling its (worrying) potency, it could also be interpreted as communicating safety. Discourses of minimisation and comparison have been found in some US research (e.g. Cutler, 2014; DeSantis & Hane, 2010) when users account for their consumption practices; similar patterns were evident in our study, with coffee frequently deployed as a comparison point to signal the safety and efficacy of modafinil (and hence to legitimate its use). Associated with rhetorical minimisation, users leveraged experiential, bodily knowledge about the effects of study drugs (‘lay pharmacology’; Webster et al., 2009) to account for their general use of study drugs and their specific patterns of consumption, as well as, more formalised pharmacological and wider knowledge. Other research has indicated a lack of risk-awareness and formal pharmacological understanding on the part of users (DeSouza, 2015; DeSantis et al., 2010). Our attention to an elucidation of the leveraging of different forms of knowledge by study drug users suggests alternate ways of exploring understandings and perspectives on risk and modes of legitimisation by study drug users.

Students reported intensified stress and anxiety levels around exam times, and accounted for these as altering how risks and benefits were weighed, shifting student’s assessments over time. The students we interviewed similarly presented the standards they had to meet to secure particular futures for themselves as set higher than for previous generations, and implied that an inability to meet these as a personal deficit that needed to be overcome, including via the use of drugs. Our respondents also spoke of being more willing to compromise on initial risk-assessments in order to complete pressing workloads. This finding aligns with research emphasising the pursuit of productivity underlying study drug use and claims that the progressive pharmaceuticalisation of society (Williams et al., 2011) is now extending into higher education (Petersen, Norgaard, & Traulsen, 2015; Vargo & Petroczi, 2016). Individuals increasingly conceive the ability to stay awake and focus as being amenable to pharmacological manipulation (Coveney et al., 2009), with sleep being customisable (Williams et al., 2013) in order to meet desired goals. Hogle (2005: 697) has described demands ‘for a new type of body’ that ‘needs little sleep and can work harder’ (see also Collin, 2016). While our data does not enable us to make clear statements about whether (or not) theories about the pharmaceuticalisation of higher education are supported, it is certainly the case that (a) study drugs are now part of the (longstanding) array of strategies used by at least some students to customise sleep(iness) and wakefulness, and (b) the pressures of university life are a readily available resource for constructing narratives of legitimation for drug use. Further, it was the facility of study drugs to modulate sleep and sleepiness that tended to be emphasised over any generalised ‘cognitive enhancement’ (i.e. drugs seem largely positioned to help students work harder, but necessary to be ‘smarter’).

To summarise, the findings of this study have augmented the existing literature examining student perspectives on ‘study drugs’, reinforcing existing claims about the extent to which drug practices are linked with situational pressures (DeSantis et al., 2010; Eickenhorst et al., 2012; Forlini & Racine, 2009b, Forlini et al., 2015; Hildt et al., 2014; Hupli et al., 2016; Partridge et al., 2013), and underscoring how consumption is largely aimed at customising sleep and sleepiness (Williams et al., 2013) and not necessarily the enhancement of cognition *per se*. We have additionally drawn attention to how lay pharmacology (Webster et al., 2009) and pharmaceutical competences (Rönkä & Katainen, 2017) are developed and deployed to legitimate and account for - and likely shape - drug use.

### Implications

Given our findings, we consider that there is at least the potential for UK educators and institutions of higher education to consider policy in relation to study-related pharmaceutical practices. However, we are also mindful of Forlini et al. (2015) scholarship, which presents a ‘challenge [to] the assumption that policy on neuroenhancement is necessary for academic environments’ (Forlini et al., 2015: 83). If any endeavours are advanced by institutions (e.g. in terms of harm reduction or health education policies) - and we are not necessarily advocating that they should be - we feel strongly that education leaders need to refrain from an exclusive emphasis on the individual and personal practices of undergraduates. Instead, they need to focus on the temporalities of university life that generate moments of heightened anxiety and stress of the kind that our respondents account for as enjoining study drug use. Infrastructural innovations in this regard might include the restructuring of assessment timetables in order to flatten out some of the peaks and troughs of examinations and deadlines throughout the academic year. In essence, any initiative geared towards intervening in study-drug use should not be divorced from wider endeavours to enhance student mental health and wellbeing (see relatedly Jensen et al., 2016). Moreover, universities must refrain from individualising student healthcare such that they undergraduates are regarded as the exclusive guardians of their own wellbeing within structures of high education that can act as impediments to health and welfare.
